# Collaborative care model for treatment of persistent symptoms after concussion among youth (CARE4PCS-II): Study protocol for a randomized, controlled trial

**DOI:** 10.1186/s13063-019-3662-3

**Published:** 2019-09-18

**Authors:** Carolyn A. McCarty, Douglas Zatzick, Teah Hoopes, Katelyn Payne, Rebecca Parrish, Frederick P. Rivara

**Affiliations:** 1 Seattle Children’s Research Institute, P.O. Box 5371, M/S: CW8-5, Seattle, WA 98145-5005 USA; 2 Department of Pediatrics, University of Washington, Seattle, USA; 3 Department of Psychiatry and Behavioral Sciences, University of Washington, Harborview Medical Center 325 9th Avenue, Box 359911, Seattle, WA 98104-2499 USA

**Keywords:** Collaborative care, Concussion, Cognitive-behavior therapy, Randomized trials, Adolescent health, Sports injury

## Abstract

**Background:**

Currently, there is limited evidence to guide intervention and service delivery coordination for youth who suffer a concussion and subsequently experience persistent post-concussive symptoms (PCS) (Lumba-Brown et al. JAMA Pediatr 172(11):e182853, 2018; Lumba-Brown A et al. JAMA Pediatr 172(11):e182847, 2018). We have developed a collaborative care intervention with embedded cognitive-behavioral therapy, care management, and stepped-up psychotropic medication consultation to address persistent PCS and related psychological comorbidities. The CARE4PCS-II study was designed to assess whether adolescents with persistent symptoms after sports-related concussion will demonstrate better outcomes when receiving this collaborative care intervention compared to a usual care (control) condition.

**Methods/design:**

This investigation is a randomized comparative effectiveness trial to receive intervention (collaborative care) or control (usual care). Two hundred sports-injured male and female adolescents aged 11–18 years with three or more post-concussive symptoms that persist for at least 1 month but less than 9 months after injury will be recruited and randomized into the study. The trial focuses on the effects of the intervention on post-concussive, depressive, and anxiety symptoms measured 3, 6, and 12 months after baseline.

**Discussion:**

The CARE4PCS II study is a large comparative effectiveness trial targeting symptomatic improvements in sports injured adolescents after concussion. The study is unique in its adaptation of the collaborative care model to a broad spectrum of primary care, sports medicine, and school settings. The investigation incorporates novel elements such as the delivery of CBT through HIPAA complaint video conferenceing technology and has excellent widespread dissemination potential should effectiveness be demonstrated.

**Trial registration:**

ClinicalTrials.gov, NCT03034720. Registered on January 27, 2017.

**Electronic supplementary material:**

The online version of this article (10.1186/s13063-019-3662-3) contains supplementary material, which is available to authorized users.

## Background

Sports-related concussions are endemic among children and adolescents and constitute a major public health challenge, particularly when they fail to resolve in a timely manner. Estimates of sports-related concussions in youth range from 1.1 to 1.9 million per year in the United States [3]. While symptoms from sports-related concussion normally resolve spontaneously within days to weeks following injury, an estimated 14% or more of school-aged children experience significant morbidity that lasts for several months [4]. Persistent post-concussive headache, fatigue, dizziness, and inattention confer marked functional impairment for affected youth and can significantly interfere with academic performance and social functioning [5, 6]. Psychological symptoms, including depression and anxiety, commonly accompany post-concussive symptoms (PCS) [7]. Symptoms of depression correlate with other post-concussion symptoms [8] and may further prolong recovery from primary symptoms, as has been demonstrated in patients with traumatic brain injury [7, 9–12]. Furthermore, recommended activity and school restrictions may contribute to increases in depression and anxiety [13]. Taken together, the complexities of persistent PCS in conjunction with co-morbid psychological symptoms create a significant burden for the injured youth, their families, and schools.

Currently, there is limited evidence to guide intervention and service delivery coordination for sports concussion-exposed youth [1, 2, 14–16]. Current clinical paradigms for persistent PCS emphasize education about symptoms, anticipatory guidance around return to physical and cognitive activity, and reassurance of a full recovery [17]. While these guidelines are adequate for patients with typical recovery, providers of patients who experience chronic symptoms are left with few treatment options because the evidence base for treatment of youth with persistent PCS is lacking [18]. Psychotherapy and medication management services can be difficult to access and are not incorporated into the standard of care [19]. Thus, healthcare providers face a major challenge in managing patients with persistent post-concussive symptomatology.

Collaborative care has demonstrated effectiveness in treating chronic disorders where both physical and psychological symptoms are present, and may be well-suited to address service delivery coordination among sports injured youth. Collaborative care is a healthcare delivery model that integrates medical and mental health and allows for individuals to receive care that is titrated to align with their clinical needs. Large-scale randomized clinical trials have established the effectiveness of collaborative care interventions that combine care management, evidence-based pharmacotherapy, and CBT in treating adult primary care patients with depressive, anxiety, and post-traumatic stress disorders [20–23]. Other studies have documented the effectiveness of collaborative care interventions in reducing anxiety symptoms and improving functional impairments in injured adults after acute injury [24–26]. A series of investigations have now established the feasibility and effectiveness of the circumscribed delivery of collaborative care for pediatric patients in primary care settings [27–30]. Fewer investigations have successfully targeted anxiety and depressive symptoms in acutely injured youth [31]. Given the constellation of somatic, cognitive, and emotional impairments associated with persistent PCS, an integrated approach to symptom management which targets these impairments in combination holds promise for youth [32–35].

We have developed a collaborative care intervention with embedded cognitive-behavioral therapy (CBT), care management, and stepped-up psychotropic medication consultation to address persistent PCS. Our pilot data showed that this intervention had large, clinically meaningful effects on reducing persistent PCS and increasing quality of life for adolescents compared to usual care [36]. Collaborative care interventions may be optimal for youth suffering from PCS presentations as these models can facilitate linkages across primary care pediatric, rehabilitation, specialty, and school-based service delivery sectors.

CBT is a robust evidenced-based treatment for psychiatric disorders including depression and anxiety; an emerging literature also supports CBT as an efficacious intervention for PCS [36, 37]. Cognitive-behavioral therapy has shown response rates of 60–80% among youth aged 12–17 years with depression and among youth aged 7–17 years with anxiety disorders, with long-term outcomes equivalent to anti-depressant medication [38–40]. Across injured and other trauma-exposed populations, CBT is the evidence-based treatment modality for anxiety most consistently recommended by best-practice treatment guidelines [41–44]. CBT has also been successfully delivered by phone and video conferencing [32, 36]. Initial studies suggest that CBT intervention strategies can reduce persistent traumatic brain injury (TBI) symptoms, including memory impairment, difficulties with concentration, and somatic symptoms such as fatigue, sleep problems, and irritability [45].

An emerging evidence base suggests that pharmacological interventions may be an effective adjunctive tool in combination with psychotherapeutic interventions in the management of PCS, anxiety, and depressive symptoms in youth and adults [39, 46–53]. The selective serotonin reuptake inhibitor (SSRI) and serotonin norepinephrine reuptake inhibitor classes of anti-depressant medication can be delivered safely to adolescents and young adults suffering from anxiety and depression with appropriate psychiatric oversight [31, 46]. Post-injury pharmacotherapy can target non-specific persistent TBI symptoms such as insomnia [25, 26, 31]. Some, but not all, investigations suggest that post-concussive headaches may be effectively targeted with pharmacotherapy [54].

**Fig. 1:**
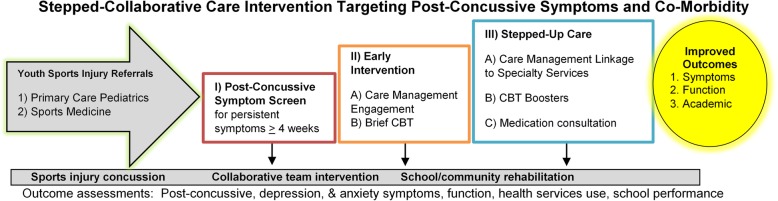
CARE4PCS-II Collaborative Care Intervention model

Intervention models that bridge primary care, community, and specialty care services in order to deliver evidence-based treatments are a crucial element of the integration of healthcare after sports injury in youth [1, 55] (Fig. 1). Intervention models that serve to link acutely exposed youth to evidence-based treatments have not been widely implemented and represent a crucial next step in adolescent sports injury intervention development [55]. The aim of this manuscript is to describe the protocol for the CARE4PCS II study in Seattle, WA, USA.

### Objectives and hypothesis

This study was designed to assess whether adolescents with persistent symptoms after sports-related concussion will demonstrate better outcomes when receiving a collaborative care intervention compared to usual care (control) condition. Four main aims are addressed; first, we want to compare the groups with respect to post-concussive, anxiety, and depressive symptoms at 3, 6, and 12 months follow-up. Second, we seek to examine the effectiveness of the intervention in improving function and health-related quality of life among adolescents with persistent symptoms after sports-related concussion. Third, the study will also evaluate the impact of the collaborative care intervention on school performance. Finally, we aim to explore the heterogeneity of treatment effects in outcomes by examining the interaction of the treatment effect with group membership in distinct subgroups of the population.

We hypothesize that adolescents who receive the collaborative care intervention will demonstrate clinically and statistically significant reductions in PCS and depressive and anxiety symptoms over the course of the 12-month study compared to the usual care control group. We also hypothesize that adolescents who receive collaborative care will exhibit a clinically meaningful improvement in functioning and health-related quality life compared to the usual care control group. For the third aim, we expect that adolescents who receive the collaborative care intervention will receive individualized treatment and community resource linkages which will improve their school performance and return to full activities at school compared to usual care. Finally, we anticipate that three distinct subgroups will emerge from the study population—adolescents who recover from symptoms, adolescents with chronic psychosocial problems, and adolescents whose symptoms wax and wane over time—and these groups will have heterogeneity in treatment effects.

## Methods/design

### Design

**Fig. 2:**
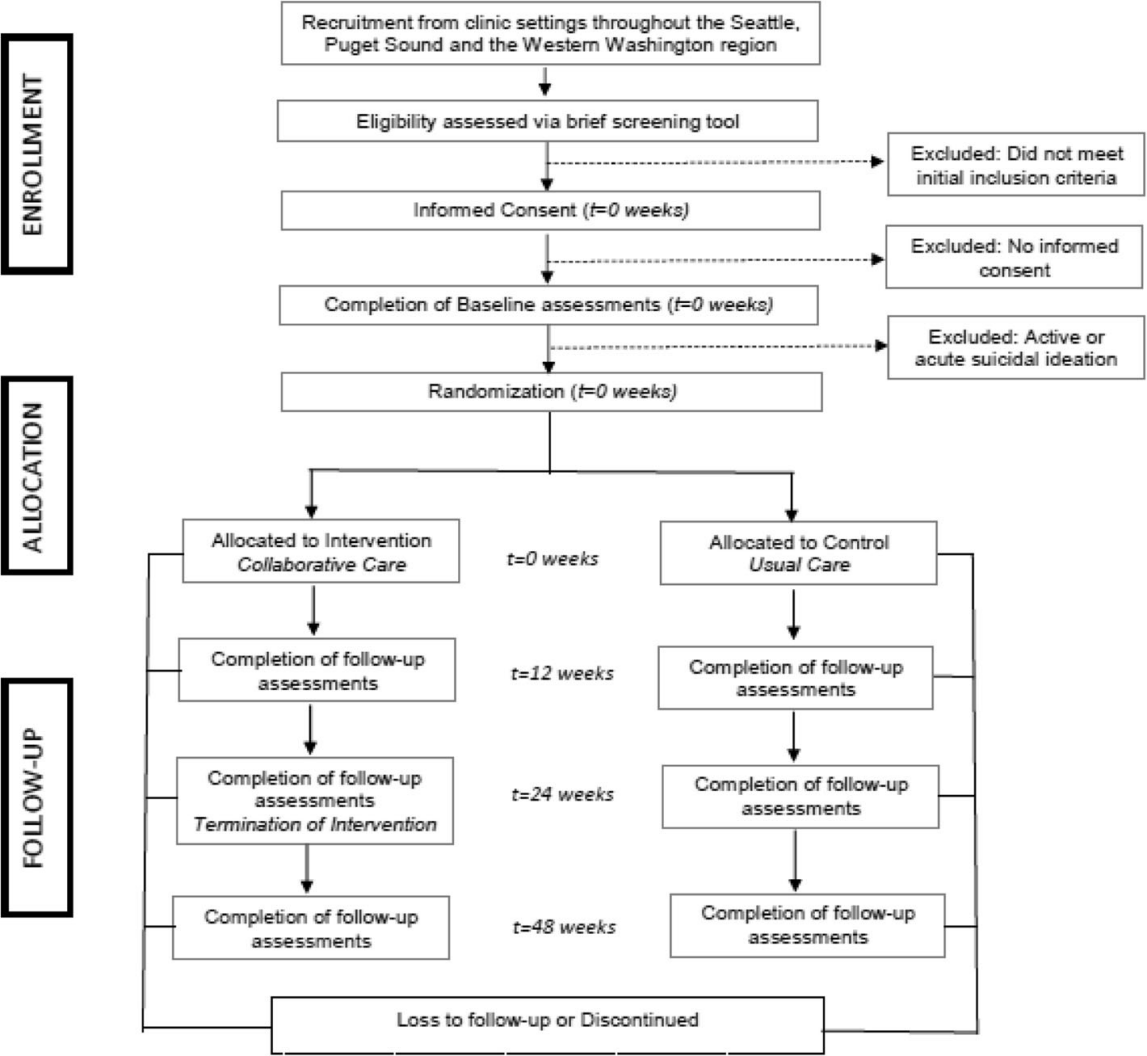
Consort trial flow diagram

This investigation is a randomized comparative effectiveness trial allocated in a 1:1 ratio to receive intervention (collaborative care) or control (usual care). The trial focuses on the effects of the intervention on youth outcomes 3, 6, and 12 months after baseline. Figure 1 shows the intervention model and Fig. 2 shows recruitment and flow of participants through the study.

### Trial setting

Subjects will be recruited from geographically dispersed primary care medical pediatric clinics, sports medicine specialty clinics, pediatric neurology clinics, and rehabilitation medicine clinics throughout the Seattle, Puget Sound and the Western Washington area of the USA.

### Participants and procedures

#### Eligibility criteria

Two hundred sports-injured male and female adolescents aged 11–18 years with at least three PCS that persist for at least 1 month but less than 9 months after injury will be recruited and randomized into the study if they and a parent can read and speak in English. The diagnosis of concussion will be made by a qualified medical provider. Adolescents with prior concussions will not be excluded. There will be active recruitment by weekly scanning of the appointment lists for the Sports Medicine, Neurology, Neurosurgery, and Rehabilitation Medicine clinics at Seattle Children’s Hospital main campus and four satellite locations, and the Sports Medicine and Rehabilitation Medicine clinics at Harborview Medical Center. Some of these settings serve patients from surrounding states, who will also be eligible for this trial.

Adolescents who have suffered spinal cord or other severe injuries that prevent participation will be excluded from the study. Adolescents will also be excluded if they have had a diagnosis of schizophrenia or psychosis, or present with active and acute suicidal ideation. Parents of adolescents who report concerns about their child’s ability to communicate may be excluded from the study pending consultation with principal investigators.

#### Informed consent

All study procedures were approved by the Seattle Children’s Hospital institutional review board (IRB; protocol number STUDY00000437) prior to the initation of participant recruitment.The research assistant or study coordinator will give each parent a brief overview of the study and ask the potential parent participant whether or not they would be interested in hearing a more detailed description and learning if their child might be eligible. If the potential parent participant wants to learn more, they will be asked to complete a brief eligibility screening tool. If the adolescent meets eligibility criteria and the parent still wants to learn more about the study, the research assistant or study coordinator will give a copy of the consent form to the parent and review the entire form with them, allowing participants to ask questions or voice concerns. The recruitment procedure will also include discussion of payment for study participation over the course of the year after the injury. Parent participants will also prospectively consent to the release of school records. After parent consent, the eligible adolescent will go through a similar study assent procedure.

There is minimal risk to subjects from data collection procedures, but the small risks that are present include the potential for distress, invasion of privacy, an accidental breach of confidentiality, and the inconvenience of participating. Significant risks to the health of subjects are low, given that the intent of the study is to compare usual care with an enhancement of usual care that is expected to be effective and safe. Upon enrollment, parent and adolescent participants will be given a study information sheet with the study follow-up survey timeline and phone number. All subjects will be in contact by phone with the study research assistants, and intervention subjects will be in frequent contact with their study care manager. Participants will be asked to notify the study personnel if any difficulties arise.

### Randomization

Randomization will occur in a 1:1 ratio, in blocks of four to six patients, according to a computer-generated random assignment sequence prepared by the study biostatistician. Once generated, intervention and control group assignments will be entered into a password-protected tracking system with access limited to study coordinator, care manager, and principal investigator. Randomization will be conducted by the study coordinator. After randomization occurs a letter and email will be sent to the referring provider and participant family to notify them as to which group the participant has been randomly assigned.

### Intervention

The collaborative care intervention includes up to three intervention components, as detailed below: 1) care management, 2) cognitive-behavioral therapy (CBT), and 3) stepped up medication consultation, if warranted. All care management and CBT are delivered by one of two study care managers on the study, both of whom are master’s level trained mental health professionals. Medication consultation is provided by a child psychiatrist on the basis of information provided by the care manager and the supervisory team; however, all prescriptions are ultimately managed by the adolescent’s existing primary care or specialty providers.

The components of the intervention can be delivered through in-person visits, HIPAA compliant video conferencing, or phone call. Zoom Video Conferencing software meets the HIPAA compliant criteria and is used with families that choose to receive the intervention in this manner. At the beginning of the intervention, families are offered this option to meet with the care manager for both care management and CBT sessions. Zoom enables care managers to meet with families that are unable to meet in person regularly. If families choose Zoom, care managers provide directions and support about how to connect by computer or by smartphone/tablet. Connecting by computer requires a camera, microphone, and internet connection. Families will be required to download Zoom software to their computer. Connecting by smartphone/tablet requires an internet connection or the ability to use cellular data. Families are required to download the Zoom application through their app store.

#### Care management

The care manager will call the family to set up an initial meeting focused on eliciting the adolescent’s and family’s concerns and treatment needs and preferences [56, 57]. The care manager will share their study cell phone number and encourage calls for questions and concerns both from the adolescent and parent participants. She will schedule ongoing times to meet the adolescent during the initial days and weeks post-randomization.

Care management meetings with subjects can occur in-person, by HIPAA compliant video conferencing, or phone call. The care manager will elicit information to formulate a comprehensive post-injury treatment plan, integrating the adolescent’s family members whenever feasible and acceptable to the adolescent. Initial discussions between the care manager and the adolescent and family members may also highlight any issues and concerns related to return to sports or academic activity.

The care manager acts as an advocate for the adolescent and family based on their needs and presenting concerns. If the adolescent or family member discloses difficulties related to academics, the care manager will contact and coordinate care with school administration. If previous or new mental health challenges arise, the care manager will support the family in finding an appropriate outside provider. The care manager will reach out to this provider to coordinate and confirm appropriate care. They will also present the plan, issues that occur during the process, and feedback from the family in the weekly intervention team meeting.

Standardized instruments will be used to monitor post-concussive, anxiety, and depressive symptoms at regular post-injury intervals. The intervention includes a motivational interviewing element embedded within care management that targets both treatment engagement and high risk behaviors that threaten recurrent injury. The motivational interviewing intervention element consists of a graded sequence of clinical tasks, including: a) eliciting from adolescents their views of the importance of changing, and of their confidence in being able to change behaviors, b) giving adolescents personalized feedback, and c) clarifying the adolescent’s behavior change goals and action plans.

#### Cognitive-behavioral therapy

The intervention includes modular CBT targeting post-concussive, anxiety, and depressive symptoms. In this CBT treatment, the adolescent is taught coping skills, relaxation strategies, and cognitive strategies to manage their symptoms, while they are encouraged to increase appropriate activation, including pacing of activities. The treatment manual is intentionally flexible to allow for tailoring to the unique presentation and needs of the adolescent, including how many sessions and what specific modules of content to deliver. CBT sessions will be tailored to adolescent preferences and may occur either in-person, by HIPAA compliant video conferencing, or phone call.

The first session involves psychoeducation and goal setting to collaboratively develop an individualized intervention plan and behavioral targets with the adolescent and their parent. Specific CBT intervention elements covered in subsequent modules may include: pain management, problem-solving, behavioral activation, mindfulness, challenging negative thinking, relaxation and imagery, avoidance, emotion regulation, family communication skills, parent and child interaction, sleep hygiene, or brief crisis intervention support. For adolescents who demonstrate adequate CBT readiness/motivation, homework assignments are given [25, 58].

Treatment is delivered primarily in individual sessions with adolescents, although parent involvement in the treatment is strongly encouraged, with the following goals: 1) educating parents about concussion symptom management, 2) creating common goals and a framework for reaching those goals, and 3) understanding the parental role in support and stress processes. The care manager monitors symptoms (depression, anxiety, PCS) on a session-by-session basis using standardized instruments to inform how treatment is working and whether to increase the intensity of CBT or consider a psychiatric consultation regarding medication initiation or changes. Adolescents may discontinue CBT when their symptoms have abated or if they feel that additional sessions are not needed/would not be helpful.

#### Medication consultation

The stepped-up medication consultation aims to initiate and ensure adequate follow-up of psychopharmacological treatment targeting PCS as well as anxiety and depressive symptoms. In the current stepped care protocol, CBT is the first treatment of choice. For patients that do not adequately respond to the combination of CBT and care management, medications become a stepped-up care option particularly when it appears that there are recalcitrant post-concussive, anxiety, or depressive symptoms that would not readily diminish without initiation of pharmacotherapy. Persistent headaches and/or insomnia can also be important treatment targets for medications when problematic and persistent. Patients who enter the intervention arm of the protocol and are already prescribed psychotropic medications will be prioritized for medication consultation.

The stepped care medication consultation option will start with a brief report from the care manager regarding symptom patterns over time; the adolescent’s current injury status; current and past comorbid medical conditions; current primary care, sports medicine and rehabilitation service use; current medication use including analgesics and psychotropic medications; prior psychotropic medication use; and attitudes and beliefs surrounding medication treatment [25, 31, 46].

For adolescent patients who have never received prior treatment for depression or anxiety, a SSRI or newer non-SSRI antidepressant is the treatment of choice [59–65]. Recommendations for medications will be made to the adolescent’s primary care physician and specialty care provider who is managing the concussion. In our prior Care for Post-Concussive Symptoms pilot study, adolescent care providers including pediatricians and treating physicians in rehabilitation medicine were often the primary prescribing providers [36, 46]. If negative side effects are reported by the adolescent, the care manager will tell the family to contact their prescribing physician as soon as possible. The care manager will also bring these concerns to the attention of the study’s child psychiatrist for consultation within 3 days, to seek recommendations for medication change or discontinuation, which will then be conveyed to the prescribing provider. The study team has expertise in the provision of long-distance pharmacological consultation, thus enhancing the reach of the current intervention in comparison to the smaller scale pilot study.

### Usual care for control subjects

Control subjects will receive usual care. Usual post-injury care may include the routine use of sports medicine, rehabilitation medicine, physical therapy, primary care emergency department, and specialty mental health services. Usual care has been selected for controls as it remains the optimal comparator condition for policy guidelines and represents care now being delivered in specialty clinics across the USA. Usual care does include referral to specialty providers caring for adolescents with concussion as needed. All aspects of concomitant care are permitted during the trial following enrollment across study arms. For control group participants who indicate suicidal ideation on follow-up surveys, a clinical assessment is conducted by a study principal investigator by telephone, with appropriate follow-up, discussion with a parent/guardian, and provision of referral resources if needed based on risk assessment.

#### Masking of treatment allocation

Study group assignments will be kept in sequentially numbered opaque envelopes that will be opened by unblinded study staff after baseline assessments to ensure allocation concealment of the randomization group. Research assistants conducting all baseline assessments and follow-up interviews will be blinded to block sizes and remain blinded to study group assignment throughout the study. Since the study team includes both unblinded (principal investigators, study coordinator, care managers) and blinded (research assistants) staff, unblinding of the research assistants is not ever necessary, although it could inadvertently occur through participant disclosure.

#### Data management

Data from the study will be derived from subject responses to questionnaires, school records, and medical records. All data will be collected specifically for research purposes. Individually identifiable private information will be collected from research participants. To ensure subject confidentiality, all research materials will be kept in a locked file cabinet in a badge-only accessible research unit.

All medical record information and other electronic data will be stored on HIPAA-compliant password-protected computers and encrypted. After data are collected, information which would identify the subjects will be removed and code numbers used instead. A study code will be assigned to each subject. All subject data will be linked to the study codes in one master file that will be encrypted and stored on a secure laboratory study computer configured behind the departmental firewall. Study data will be collected and managed using REDCap (Research Electronic Data Capture) electronic data capture tools hosted at the University of Washington [66]. REDCap is a secure, web-based application designed to support data capture for research studies. All data will be stored for 5 years after completion of the study, and only the study team will have access to the data. Additionaly, a bi-annual data quality report is produced (and shared with the DSMB) to assess quality of data collected. During these routine data quality audits, an unblinded research staff assesses missingness of data, performs range checks for data values, and conducts source data verification of electronic data.

### Quality control procedures

#### Training research assistants

During the start-up phase of the protocol, the research assistants will be trained to a reliable standard with the assessment procedures through: 1) attendance of practice training sessions on survey outcome assesment and procedures in accordance with Good Clinical Practice guidelines, and 2) individual supervision and shadowing with lead clinical research staff.

#### Training and supervising care management team

During the start-up phase of the protocol, the intervention team members will be trained in the collaborative care intervention through observation, shadowing, reading, review, and discussion of the intervention manual. All CBT treatment cases will be supervised weekly by CM, a licensed clinical psychologist who developed the modular treatment. In addition, weekly collaborative care team meetings will be used for ongoing case management. The care managers will meet weekly to staff cases with the study psychologist, psychiatrist, and pediatrician. The study team will incorporate specialized pediatric pharmacologic supervision on an approximately every other week basis.

#### Fidelity to the intervention treatment model

Care managers document the intervention elements delivered to each participant, including care management and CBT modules, as well as time spent and who was involved, on a session-by-session basis using REDCap. As an additional quality check, a subset of CBT sessions will be audio-recorded and independently coded for adherence to the intervention manual.The lead psychiatry investigator (DZ) completes medication consultation notes in REDCap for participants who are discussed during the bi-weekly team meetings.

### Baseline measures

Parents will be asked to describe family demographic characteristics including marital status, ethno-cultural heritage, and number of children. Caregivers’ education levels and combined family incomes will also be obtained and used as a measure of family resources. We will collect information on prior history of treatment for psychiatric disorder, prior and current use of psychotropic medications, prior PCS, and use of other healthcare services both by means of parent questionnaire and medical record review [25, 67].

### Outcome evaluation

For all adolescents enrolled in the trial, follow-up surveys will be completed online or by telephone at 3, 6, and 12 months after the traumatic injury, with monetary incentives for each. Participants will be invited to complete follow-up surveys even if they have discontinued or not fully engaged in the intervention protocols. Research assistants will be trained to bring up specific discussion points about the benefits of study participation in cases where control group families are unsure about continuing their participation, to increase their retention and counteract differential dropout. Table 1 provides a summary of data collection time points. The study includes a variety of outcome measures to cover the wide gamut of issues experienced by youth with persistent post-concussive symptoms, such as depression, anxiety, sleep interference, headaches, and poor school performance.

**Table 1:** List of study measures by timepoint

Construct	Measure	Screen	Base-line	3 months	6 months	12 months
History of TBI		P	P			P
Post-concussive symptoms	HBI	A, P	A, P	A, P	A, P	A, P
Depressive symptoms	PHQ-9		A, P, PS	A, P, PS	A, P, PS	A, P, PS
Anxiety symptoms	Generalized Anxiety Disorder-7 item, revised Child Anxiety and Depression Scale		A, PS	A, PS	A, PS	A, PS
Quality of life	Peds Quality of Life		A, P	A, P	A, P	A, P
Exposure to life stress–adolescent	UCLA Reaction Index Trauma History, at baseline; Life Events Checklist–Child Form at 6 and 12 months		A		A	A
Exposure to life stress–parent	National Comorbidity Study –Trauma History measure at baseline; LES at 6 and 12 months		PS		PS	PS
Sleep	Adolescent Sleep-Wake Scale		A	A	A	A
Headache	TBI-QL–Headache Pain		A	A	A	A
School attendance and performance	Questionnaire and school records		P, SR	P	P	P, SR
Satisfaction with care	Client Satisfaction Questionnaire				A, PS	
Prior psychiatric history	Questionnaire		P			
Demographic characteristics	Questionnaire		PS			
Health Service and Medication Utilization	Questionnaire		P	P	P	P

*A* adolescent, *P* parent report of youth, *PS* parent self-report, *SR* school records, *TBI* traumatic brain injury

#### Primary outcome measures

##### Health Behavior Inventory

The Health Behavior Inventory (HBI) is a 20-item questionnaire that assesses PCS on a four-point scale, ranging from “never” to “often”, and yields total scores in cognitive and somatic domains. The scale includes youth-report and parent-report versions with established reliability and validity in youth with sports injury [68].

##### Patient Health Questionnaire

We will use the nine-item Patient Health Questionnaire (PHQ-9) to measure severity of depressive symptoms. Reliability and validity of the PHQ-9 have been established in pediatric populations [69]. Parental self-report of depressive symptoms will also be measured using the PHQ-9.

##### Anxiety measures

We will use the Generalized Anxiety Disorder-7 item scale [70] and the 15 anxiety items of the Revised Child Anxiety and Depression Scale [71] for adolescent report on anxiety and parent report on adolescent anxiety.

#### Secondary outcome measures

##### Pediatric Quality of Life Inventory

The Pediatric Quality of Life Inventory (PedsQL) is a 23-item questionnaire that assesses physical, emotional, social and school functioning. The scale includes youth-report and parent-report versions [72, 73].

##### School performance

Parents will complete the eight-item Concussion Learning Assessement and School Survey to measure post-injury academic experiences, including number of days missed, concerns about school performance, impact on grades, and whether the school provides academic support to kids with concussion [74]. This will be repeated at each of the follow-up assessments. Participants’ 1 year pre- and post-injury school attendance and grades will also be obtained from school records as a measure of academic outcomes [31] and will be re-coded using a standardized four point grade-point average (GPA).

#### Other outcome measures

##### Client Satisfaction Questionnaire

The eight-item Client Satisfaction Questionnaire will be used at 6 months to measure adolescent and parent satisfaction with services; it has excellent correlation with changes in symptom score [75]. An open-ended question was added to the end of the client satisfaction questionnaire, “*Please let us know if you have any other thoughts or additional comments about your experience with the care you received during the study*,” to allow participants to share their experiences of the intervention or of the usual care received.

##### Adolescent Sleep Wake Scale (ASWS)

The ten-item version of the Adolescent Sleep Wake Scale (ASWS), including domains of falling asleep, reinitiating sleep, and returning to wakefulness, will be used as a measure of sleep quality [76].

##### TBI-QOL–Headache Pain

TBI-QOL-Headache Pain is a 13-item tool that asks participants to estimate how often they experience different issues associated with headaches and headache pain using a Likert scale [77, 78].

#### Health service and medication utilization

The Zatzick et al. [25] and MacKenzie et al. [79] Parent report will be used to assess adolescents’ pre- and post-sports injury health service utilization. Parents will report on emergency department and outpatient pediatric and any specialty sports medicine care. Parents will also report on types of medication used by adolescents. This will be supplemented by information from the medical record.

#### Moderators of treatment outcome

##### History of sports concussion

Adolescents and parents will provide a retrospective report of previous concussion events or concussion-like symptoms they experience. This assessment will include time of previous concussion events, mechanism of injury, and resulting onset and duration of symptoms. An additional set of questions regarding re-injury during the study time period will be asked in the 12-month survey.

##### Exposure to life stress

For adolescent subjects we will use the UCLA Reaction Index Trauma History to collect information at baseline [80]. This measure asks whether or not the respondent has ever experienced ten specific and different traumatic events. The Life Events Checklist will be collected at the 6- and 12-month time points, asking the respondent about whether or not specific life events occurred in the past 6 months, if the event was good or bad, and the level of effect the event had on the respondent’s life. For parent participants we will use an adapted version of the questionnaire used in the National Comorbidity Study–Trauma History at baseline [81]. This will ask about prior abuse, assault, witnessing or experiencing an accident, disaster, life threating illness, death of loved ones. At 6- and 12-month time points we will use the Stressful Life Events Scale, which asks whether or not a parent has experienced specific events [82].

### Data analysis

#### Data analysis plan and statistical procedures

Descriptive statistics for demographic variables, symptom levels, and functional status will be tabulated by the project statistician. All scales will be scored and subscales described. This process will include examining the data for missing values, appropriate ranges and outliers, as well as construction of consort flow diagrams. All primary statistical analyses will be conducted with the intent-to-treat sample.

The primary purpose of the statistical analyses is to examine and compare trends in quality of life and post-concussive, anxiety, and depressive symptoms longitudinally between adolescents in the intervention and control conditions. The effect of major interest will be the time-by-treatment group interaction term. We hypothesize that intervention adolescents will demonstrate greater improvement than controls in both self-report and parent report of post-concussive, anxiety, and depressive symptomatic and quality of life outcomes over the course of the year of trial participation.

We will use mixed random effects generalized regression models to test this hypothesis for continuous and discrete outcomes [83–86]. These models permit the inclusion of subjects with missing data and allow for individual varying slopes and intercepts over time. In addition, these models will allow use of covariates to model potential sources of non-response bias and time-dependent covariates. This model also allows the specification of random or fixed effects and the form of the serial correlation over time (if heterogeneity changes over time). Prior to these analyses, we will examine baseline group differences using the appropriate statistics for the distribution of the variable. Although randomization should ensure balance between the two groups, it is essential to control for known confounders in the design and analysis to prevent a biased assessment of the treatment effect.

Baseline injury, demographic, or clinical variables found to be statistically significant in this analysis will also be included as covariates in the regression models. Some attrition is expected in the study samples. In prior randomized trials of injured adolescents, the investigative group has achieved 6–12-month follow-up completion rates ≥ 90% [31]. Estimates derived from these rates are incorporated into descriptions of subject flow and power analyses. Assumptions about the nature of missing data are crucial to the type of statistical analysis chosen [87–89]. Full information maximum likelihood estimates from mixed random effects generalized linear models adjust for data missing at random (MAR) [90].

We will use statistical logistic models to determine which, if any, demographic or clinical characteristics, including treatment group membership, are predictive of subject attrition. Any factors observed to explain trends in missing data would be used as covariates in subsequent analyses. In past studies, no sources of consistent variation to explain missing data were found. Based on our low attrition rates and the lack of consistent variation in past investigations, we believe that MAR is a reasonable assumption. However, we will perform a sensitivity analysis using non-MAR techniques. The analysis of missing data is an area of ongoing development, and the investigative group will incorporate the most contemporary approaches in the final analyses [88, 89].

We will test for changes in the GPA at 12 months compared to pre-injury in the intervention group participants compared to those in the control group. We will also examine differences in the scores on standardized tests in the same fashion, as well as examine for differences in time lost from school, including missing whole days as well as missing partial days. Secondary analyses will explore the associations with gender, type of sports injury, pre-injury history of psychiatric disorder or visits, and cumulative lifetime trauma history and any observed treatment effects.

We will examine heterogeneity of treatment effects in the primary and secondary outcomes by examining the interaction of the treatment effect with group membership in distinct subgroups of the population. These will be exploratory analyses. Based on our clinical experience, we hypothesize at least three groups of patients: (1) children with mild PCS who, while the symptoms last longer than for most children, nevertheless do resolve relatively quickly; (2) a group of children with persistent PCS and prior history of substantial psycho-emotional distress; (3) a group of children who have symptoms that affect their quality of life, but which wax and wane over time. We will explore defining these groups (1) a priori using clinical indicators and symptoms and (2) empirically using the study sample data, such as latent profile analysis or trajectory analysis. The analysis will define these groups and examine the heterogeneous effect of the intervention of the recovery profiles of these groups.

#### Power analysis and sample size

Power analyses were conducted using PASS software to determine the appropriate number of participants for the study [91–93]. Based in part on prior investigation by the study team, assumptions including four assessment points (baseline, 3 months, 6 months, and 12 months), equal correlations over time of ρ = 0.7, two-tailed alpha = 0.05, and 10% 12-month attrition were used for all power analyses. We used data derived from our prior large scale collaborative care investigations to estimate symptomatic and quality of life treatment effects for power calculations [25, 94]; data from the pilot were used to corroborate treatment effect estimates derived from these larger scale trials [95, 96]. The study team conservatively estimated that the 12-month persistent concussive symptom treatment effect will be d = 0.35–0.40, using preliminary data derived from prior study team large scale collaborative care investigations [25, 31].

To estimate the power to detect a group-by-time interaction effect on persistent concussive symptoms we used the primary continuous HBI total symptom score with baseline mean = 33.0 and common SD = 11.0. The study will require recruitment of 200 subjects (100 subjects in each group) in order to retain 180 subjects at the 12-month post-injury follow-up. With a final sample of 180 subjects, the power to detect a significant group-by-time interaction with a between-group effect size of d = 0.36 on the HBI somatic symptom scale is 0.80.

The study team also estimated the power to detect between-group differences in 12-month treatment response rates based on a response cutoff level of three or more symptoms on the HBI. If at the 12-month study endpoint 44% of subjects in the intervention group vs 65% of subjects in the control group continue to meet the PCS symptomatic criterion of three or more symptoms on the HBI, the power is = 0.80. Data from the collaborative care pilot corroborate these estimates. The pilot demonstrated a 0.74 treatment effect on the 6-month symptoms measured by the HBI [36]; with 180 subjects the power to detect a significant treatment group difference is ≥ 90%. In our prior R01 study, the 12-month post-injury treatment effects for the Medical Outcomes Study Short Form 12/36 Physical Components Summary were approximately 0.30 [25]. In the current study we use a PEDS-QL study entry baseline of 59.0 and common SD of 14.4. Anticipating an effect size of d = 0.37 with 90 subjects in each group, the power to detect a significant group by time interaction effect is 0.80. The pilot study demonstrated a 0.66 treatment effect on the HBI; with 180 subjects the power to detect a significant treatment group difference is ≥ 90%.

### Data collection and security

The University of Washington (UW) Institute of Translational Health Sciences (ITHS) REDCap instance is being utilized for collection and storage of patient enrollment data, patient-reported outcomes, intervention and enhanced usual care delivery documentation, and the tracking of patients for follow-up assessments. The ITHS REDCap installation is configured to be HIPAA compliant, and is housed in the secure UW data center. The installation currently runs on two virtualized environments, one for the webserver and one for the database itself.

### Data and Safety Monitoring Board

Ongoing monitoring of the study for futility, data integrity, and safety will be conducted by an external independent Data Safety and Monitoring Board (DSMB). The board will consist of a pediatric bioethicist (who will also serve as the Board Chair), a neuropsychologist skilled in the treatment of concussion among youth, and a quantitative biostatistician.

Prior to protocol initiation, the DSMB reviewed the study procedures and plans for safety monitoring. Over the course of the trial, the board will review the recruitment and retention of participants monthly and will monitor the occurrence of adverse events. All potential adverse events will be reported within 48 h to the DSMB. These events include deaths, suicide attempts, severe medication side effects, study dropout, psychiatric hospitalizations, and clinical deterioration defined as the development of new suicidal or homicidal behaviors. The DSMB will also conduct reviews of study progression and data integrity to include assessments of attainment of study recruitment milestones, review of modifications made to the protocol, adequacy of follow-up, and threats to internal validity such as differential drop-out of subjects in the intervention versus control conditions*.* Interim analyses will be conducted in February, 2019 for the purpose of examining effects for future grants, and results will be shared with the DSMB. In this youth concussion-focused comparative effectiveness trial, either negative or positive results will serve to inform a current paucity of randomized trial evidence. Therefore, stoppage rules primarily target futility related to an inability to recruit an adequate sample size and do not extend to the results of interim analyses.

The DSMB will meet formally via teleconference every 6 months. Each year, the oversight board will produce a report that summarizes 1) all serious and unexpected adverse events, 2) the committee’s opinion as to whether safety, confidentiality, and privacy have been adequately assured by the investigators, 3) a summary of progress towards recruitment and follow-up goals. The yearly summary will be forwarded to the principal investigators, who will in turn forward the summary to the Seattle Children’s Research Institute IRB. Further details on the roles and responsibilies of the DSMB can be found within the Data Safety Monitoring Board Charter (Additional file 1).

#### Dissemination policy

The study team plans to write up study results for publication in a scientific journal article to reach healthcare professionals. Study results will also be posted on ClincialTrials.gov. If the collaborative care intervention is found to be superior to care as usual, we plan to write a grant to further develop dissemination mechanisms to train other healthcare professionals in this model. We will abide by the International Committee of Medical Journal Editors guidelines regarding authorship, and plan to provide study team members first access to the data for addressing the main objectives stated here. We do not plan to use professional writers. The final de-identified study dataset will be shared on the National Institute of Child Health and Human Development Data and Specimen Hub for the purposes of secondary research use.

## Discussion

The CARE4PCS-II study is the largest collaborative care trial to date to target symptomatic improvements in sports injured adolescents after concussion. The investigation builds upon and extends the established randomized clinical trial evidence base supporting collaborative care interventions for youth and adults presenting with medical and psychiatric co-morbidity to primary and acute care settings [20–30]. The investigation was preceeded by a smaller scale study team pilot investigation that used CBT as the primary evidence-based treatment modality in a stepped care protocol with the option to recommend psychotropic medication to injured youth with enduring post-concussive symptoms. The pilot study suggested that the stepped collaborative care intervention may be effective in reducing PCS and other co-morbid symptoms in concussion-exposed youth [36]. The study team is aware that the prioritization of CBT over medications in our stepped care protocols for concussed youth differs from previous primary and acute care adult collaborative care procedures that have given equal priority to CBT and pharmacotherapy [14, 15, 25]. The decision to make CBT the primary evidence-based treatment modality was based on a review of the adolescent CBT and pharmacotherapy literatures. Subgroup analyses examining heterogeneity of treatment effects may be required should the current trial recruit a substantial number of concussed adolescents who are on psychotropic medication at the time of their sports injury. The study is unique in its adaptation of the collaborative care model to a broad spectrum of settings in which adolescents receive treatment for the sequalae of their sports injuries. Care in the current protocol can link patients seen in multiple pediatric, primary care, sports medicine, and school settings. Prior adolescent collaborative care intervention studies have focused more narrowly on recruitment and linkage from selective primary and acute care medical settings [27–31].

The study is designed to broadly reach patients referred throughout the Seattle, Western Washington, and Puget Sound region. Multiple intervention elements support the broadening of the reach of the collaborative care treatment, including the use of HIPAA-compliant videoconferencing technology that allows for the expansion of CBT delivery beyond regional clinic settings that has previously characterized collaborative care clinical trials for youth. Similarly, pediatric psychopharmacologic intervention procedures have been adapted from statewide pediatric consultation models [46], which are unlikely to reach sports injured youth with persistent concussive symptoms without the orchestrated linkage procedures provided by collaborative care. Should the trial demonstrate effectiveness the study team is uniquely positioned to develop and roll-out nationwide dissemination efforts. The study team has an established track record of working with professional societies and federal agencies to disseminate the results of primary and secondary intervention for youth and adults [97–100].

## Trial status

The trial began recruitment of participants in March, 2017, and we expect to recruit the full sample near the end of March, 2019. The 12-month follow-up assessments of all the participants would then be completed by March, 2020. Protocol version #7; protocol version date October 26, 2018.

## Additional files

Additional file 1: Charter for the Independent Data Monitoring Committee for the trial: Collaborative care for persistent symptoms after concussion. (DOCX 66 kb)

## Data Availability

Not applicable.

## Abbreviations

ASWS: Adolescent Sleep-Wake Scale
CBT: Cognitive-behavioral therapy
DSMB: Data Safety and Monitoring Board
GPA: Grade point average
HBI: Health Behavior Inventory
HIPAA: Health insurance portability and accountability act
IRB: Institutional review board
ITHS: Institute of Translational Health Services
MAR: Missing at random
PCS: Post-concussive symptoms
PEDS-QL: Pediatrics Quality of Life Inventory
PHQ-9: Patient Health Questionnaire-9
REDCap: Research electronic data capture
SSRI: Selective serotonin reuptake inhibitor
TBI: Traumatic brain injury
UW: University of Washington
