# Supplementary material for: Collaborative care model for treatment of persistent symptoms after concussion among youth (CARE4PCS-II): Study protocol for a randomized, controlled trial
Source: Trials. 2019 Sep 18;20:567. doi: 10.1186/s13063-019-3662-3 (PMC6749638; doi:10.1186/s13063-019-3662-3)
Supplement: Supplementary file 1 — Charter for the Independent Data Monitoring Committee for the trial: Collaborative care for persistent symptoms after concussion. (DOCX 66 kb) [file 13063_2019_3662_MOESM1_ESM.docx]

**Charter for the Independent Data Monitoring Committee for the trial:**

**Collaborative Care for persistent symptoms after concussion**

**Version 1.2, February 2017**

***1. Introduction***

This document constitutes the Independent Data Monitoring Committee (IDMC) Charter for the **Collaborative care for persistent symptoms after concussion trial**, to be conducted at the Seattle Children’s Research Institute and the University of Washington*.*

This Charter is a living document and will be reviewed periodically by the IDMC to determine whether any changes in procedure(s) are needed throughout the duration of the study. All version updates to this document are tracked in the table below.

| **Version Number** | **Date Need for Change Was Identified** | **Details of Change** | **Date Change was Finalized** |
| --- | --- | --- | --- |
| 1.0 |  | Drafted Initial Charter | **12/12/2015** |
| 1.1 |  | Revision of Initial Charter draft | **1/8/16** |
| 1.2 | **2/13/17** | Named Chris Feudtner as corresponding secretary for IDMC, replacing previously described executive secretary | **2/13/17** |

Each member of this IDMC must agree to the terms outlined in this Charter. Each member will sign the *Acceptance of IDMC Terms and Conditions Form* to illustrate this agreement. Once this Charter is finalized, the final Charter will be reviewed in an IDMC meeting, signed by all members, and the signed copies provided to the NICHD Program Officer for the study.

***2. Purpose and Responsibilities of the IDMC***

The members of the IDMC identified in this Charter for the *Collaborative care for persistent symptoms after concussion trial* are responsible for safeguarding the interests of study participants, assessing the safety and efficacy of all study procedures, and shall monitor the overall conduct of the study*.* This Committee will serve as an independent advisory group to the Director of the NICHD and is required to provide recommendations about starting, continuing, and stopping the trial. The Committee will:

- Review the research protocol, review model informed consent documents, and plans for data and safety monitoring, including all proposed revisions;
- Review methodology used to help maintain the confidentiality of the study data and the results of monitoring by reviewing procedures put in place by investigators to ensure confidentiality;
- Monitor study design, procedures and events that will maximize the safety of the study participants and minimize the risks;
- Evaluate the progress of the study, including periodic assessments of data quality and timeliness, participant recruitment, accrual and retention, participant risk versus benefit, performance of the study site(s), and other factors that may affect study outcome;
- Consider factors external to the study when relevant information becomes available, such as scientific or therapeutic developments that may have an impact on the safety of the participants or the ethics of the studies;
- Review serious adverse event documentation and safety reports and make recommendations regarding protection of the safety of the study participants;
- Report to the NICHD on the safety and progress of the study;
- Evaluate and report to the NICHD on any perceived problems with study conduct, enrollment, sample size, and/or data collection;
- Provide to the NICHD a recommendation regarding continuation, termination or other modifications of the study based on the cumulative experience including the observed beneficial or adverse effects of the treatment under study;

This Committee is responsible for identifying mechanisms for the completion of various tasks that will impact the safety and efficacy of all study procedures and overall conduct of the study. The table below identifies the key areas for which oversight is necessary and the ways in which the Committee for the study will complete those tasks.

| **Basic Responsibility of IDMC** | **Method IDMC for the *Collaborative care for persistent symptoms after concussion trial* will use to complete task** |
| --- | --- |
| Familiarize themselves with the study protocol | Will read and review the study protocol provided by the investigators |
| Monitor adverse events | Will be copied on all correspondence with the IRB on adverse events |
| Monitor data quality | Will examine the quality of the data every 6 months through an analysis of data to determine missing data, erroneous values |
| Oversee participant recruitment and enrollment | Will review monthly recruitment and enrollment data and compare that to the projected enrollment |
| Develop an understanding of the Study’s risks and benefits | Will read and review the study protocol |
| Ensure the proper reporting occurs | Will be copied on annual reports to NIH |
| Review confidentiality procedures | Will be copied on IRB applications |
| Recommend study disposition | Will vote regarding recommendations to continue the study as set forth in the IRB approved protocol, or to suspend, halt, or otherwise alter the conduct of the study |

***3. IDMC Members, Organizational Chart, and Communications***

**Members**

The IDMC for the study is composed of the members listed in the table below. In addition, their high level roles and responsibilities are identified in the table.

| **Name of Member** | **Role on IDMC** | **High Level responsibilities** |
| --- | --- | --- |
| Chris Feudtner, MD, PhD, MPH | Voting member  Chair of committee  Corresponding secretary | Review the ethical aspects of the trial |
| Keith Yeates, PhD | Voting member | Review the study eligibility and intervention procedures |
| Waylon Howard, PhD | Voting member | Review the power of the study and data analysis plan; conduct interim analyses |
| NICHD Program Officer | Advisory member | Report to NICHD director |

Only voting members for this IDMC may attend closed sessions for this Committee. In addition, only voting members will have access to data for this Committee.

Chris Feudtner, MD PhD MPH, will serve as chair for the committee and as corresponding secretary (CS) for the IDMC, and will assure the accuracy and timely transmission of IDMC final recommendations and IDMC meeting minutes to the relevant parties.

**Organizational Chart**

The following diagram illustrates the relationship between the IDMC and other entities in the study.

**

**Communication**

Communication members for this IDMC will be primarily through the NICHD Program Office. Investigators from the study will not communicate directly with IDMC members about the study, except when making presentations or responding to questions at IDMC meetings or during scheduled conference calls.

***4. Conflict of Interest (COI) and Compensation***

It is extremely important that all members of the IDMC state any real or apparent COIs at the onset of the study. Members of the IDMC shall read the NICHD Clinical Research Guidance Document regarding COI and will provide their signed summary of any COIs to the ES, for the study, at its onset. Prior to each meeting, all members of the IDMC will have an opportunity to state whether they have developed any new COIs since the pervious meeting. As a new COI is identified, it must be documented and a new signed summary of the COI should be provided to the CS.

If a new conflict is reported, the Chair and staff will determine if the conflict limits the ability of the IDMC member to participate in the discussion.

All IDMC members will be compensated for their role in supporting the committee. Compensation will be $4000 over the course of the study.

***5. Scheduling, Quorum, and Organization of Meetings***

The purpose of the first meeting for the IDMC for the *Collaborative care for persistent symptoms after concussion trial* is to:

- Draft, review, discuss and sign the Charter;
- Provide an overview of study activities;
- Review and make recommendations about the study protocol(s); and
- Determine the frequency of interim analyses and whether data will or will not be masked regarding intervention assignment identity of randomized groups.

In addition to familiarizing the committee with the study at the first meeting, the IDMC will determine logistics for following meetings, which are documented in the table below.

| **Meeting / Review Type** | **Scheduled Time** | **Purpose** | **Required Attendees** |
| --- | --- | --- | --- |
| Kickoff Meeting | Prior to enrollment of study participants | - Review charter template and draft Study specific information - Identify data for review at future meetings and how it should be presented at future meetings - Review protocol including review of statistical analysis plan | All |
| Regularly scheduled conference calls | Every 6 months | To review study progress | All |
| Ad hoc Conference calls | As needed | To review any adverse events | All |
| Review of interim data analyses | Annually | To detect any pre-determined statistically significant signal of intervention benefit or harm, or any previously undetected problems with study enrollment or data quality | All |

It is expected that all IDMC members who are identified above will attend every meeting.

***7. Materials and Protocol for IDMC Meetings***

The agenda for IDMC calls will be drafted by the corresponding secretary. The IDMC Chair will review the finalized agenda prior to distribution to the group.

The agenda and meeting materials will be distributed to the IDMC before each call to allow members adequate time to prepare for the meeting. Meeting materials will include the following reports and data:

- Adverse event data
- Other safety data
- Quality and completeness of study data
- Enrollment data

The IDMC will review the above information at each meeting to ensure proper conduct of the study.

**Meeting Protocol**

IDMC calls for the study will be organized into closed and executive sessions. Definitions for each meeting type are included below. The meeting type will be identified by the DCC when it provides the IDMC Chair with the meeting agenda.

- **Closed sessions:** The IDMC and study staff will discuss confidential data from the study, including information on efficacy and safety by treatment arm.

The IDMC may decide whether to remain masked to the treatment assignments at each meeting. If the closed session occurs on a conference call, steps will be taken to ensure that only the appropriate participants are on the call and to invite others to re-join the call only at the conclusion of the closed session.

- **Executive sessions:** Only the IDMC members are present to discuss study issues independently. Voting on recommendations will follow Roberts’ Rules of Order

At the conclusion of the closed and executive sessions, all participants will re-convene so that the IDMC Chair can provide a summary of the IDMC’s recommendations. This process provides an opportunity for study investigators ask questions to clarify the recommendations. The meeting is then adjourned.

***9. Reporting Requirements for the*** Collaborative care for persistent symptoms after concussion trial ***IDMC***

Proper records will be collected at each IDMC meeting to ensure that there is a physical record of any and all decisions and recommendations. The required documentation for IDMC meetings for the *Collaborative care for persistent symptoms after concussion trial* includes the following:

- **Initial summary:** The CS is responsible for assuring the accuracy and transmission of a brief summary of the IDMC’s discussion and recommendations for the NICHD Director within 48 hours of the meeting or call. The Director or designee will review this summary and approve/disapprove the recommendation(s) or request additional information. The recommendations will then be sent to the DCC and the clinical investigators.
- **Formal minutes:** The CS is responsible for the accuracy and transmission of the formal IDMC minutes to the NICHD Director within 30 days of the meeting or call. These minutes are prepared to summarize the key points of the discussion and debate, requests for additional information, response of the investigators to previous recommendations, and the recommendations from the current meeting.
- **Action plan:** If the IDMC’s recommendations require significant changes or followup, NICHD staff and the DCC will collaborate to prepare an action plan

Minutes will be reviewed by NICHD staff, key study personnel, and the DCC before being forwarded to the IDMC Chair for final review and approval. The IDMC Chair may sign the minutes or indicate approval electronically via e-mail. Then, the minutes are sent to the NICHD Office of the Director approval. Subsequently, the minutes are sent back to the DCC and the relevant investigators and are included in the materials for the subsequent IDMC meeting to be approved by voice vote at that meeting. Once they have been voted and approved by the Board, they are considered final and archived with other IDMC documentation.

***Acceptance of Independent Data Monitoring Committee Terms and Conditions Form***

I, ____________________, member of the Data Safety and Monitoring Committee for the *Collaborative care for persistent symptoms after concussion trial***,** agree to the terms outlined in this Charter Version 1.2. If the charter is changed at any time, all Committee members will review the changes and must agree to the new Charter.

________________________ _________________________ __________

Signature Printed Name Date

________________________ _________________________ __________
Committee Chair Signature Printed Name Date

***Conflict of Interest Statement***

I, _____________, assuming the role of IDMC member for the *Collaborative care for persistent symptoms after concussion trial* agree to the following statements.

□ I agree to:

- Protect the interests and safety of study participants;
- Uphold the integrity of the research process, including data collection and analysis, to be as free from bias and preconception as I am able;
- Adhere to the highest scientific and ethical standards, comply with all relevant regulations, and eliminate or disclose, during my involvement with the proposed clinical research project, any real or apparent conflicts of interest.

In addition:

□ I declare that I, my spouse or dependent children, or organization with which I am connected, do not have any financial interest in the *Collaborative care for persistent symptoms after concussion trial*, where financial interested is defined by the U.S. Department of Health and Human Services (DHHS), as anything of monetary value, including but not limited to, salary or other payments for services (for example, consulting fees or honoraria); equity interests (for example, stocks, stock options or other ownership interests); and intellectual property rights (for example, patents, copyrights and royalties from such rights).

The financial interest term does not include various items which can be found in The Federal regulation, Public health Service (PHS), DHHS Part 50: Policies of General Applicability, Subpart F: *Responsibility of Applicants for Promoting Objectivity in Research for Which PHS Funding Is Sought.*

□ I agree not to withhold any data related to the *Collaborative care for persistent symptoms after concussion trial* or to interfere with the analysis or publication of the study’s results.

□ I will not engage in activities that could be viewed as real or apparent conflict of interest, including but not limited to:

- Having a part-time, full-time, paid, or unpaid employee status of any organizations that are: (a) involved in the study under review; (b) whose products will be used or tested in the study under review, or whose products or services would be directly and predictably affected in a major way by the outcome of the study;
- Being an officer, member, owner, trustee, director, expert advisor, or consultant of such organizations;
- Being a current collaborator or associate of the principal investigator (applicable to potential members of data safety and monitoring boards);
- Having a scientific interest beyond that required for my role, for which scientific interest is defined as having influence over the protocol, the study design, conducting the study analysis, or any reporting related to the investigation (applicable to potential members of data safety and monitoring boards).

________________________ _________________________ __________ Signature Printed Name Date

***Independent Data Monitoring Committee Confidentiality Agreement***

I understand that I will be provided with information from the Data Coordinating Center and/or study sites or similar organizations for the *Collaborative care for persistent symptoms after concussion trial* including proprietary and confidential information.

I understand that I will have access to these records in order to participate in the Independent Data Monitoring Committee for the *Collaborative care for persistent symptoms after concussion trial*

In my role as member of the IDMC, I hereby agree that I shall not release, publish, or reproduce these records. I further agree that I shall not make any use of these records except for the limited purpose of participation in the Independent Data Monitoring Committee for the *Collaborative care for persistent symptoms after concussion trial.*

I will take reasonable precautions to prevent access by any other persons to these confidential records or to work products that result from review of those records. I will retain any confidential documentation until the conclusion of the study and will return the documents and all related materials to the Executive Secretary for this study.

I have read the terms of this agreement and agree to abide by its terms.
Signed: ____________________________________ Date: ___________________

[Name], [Title]
